# Impact of Long-COVID in children: a large cohort study

**DOI:** 10.1186/s13034-024-00736-w

**Published:** 2024-04-15

**Authors:** Ziv Hersh, Yiska Loewenberg Weisband, Ariel Bogan, Adir Leibovich, Uri Obolski, Daniel Nevo, Ran Gilad-Bachrach

**Affiliations:** 1 Clalit Health Services, Tel-Aviv, Israel; 2 Clalit Research Center, Innovation Division, Clalit Health Services, Tel Aviv, Israel; 3 Department of Biomedical Engineering, Tel Aviv University, Tel-Aviv, Israel; 4 Leadspace, Hod Hasharon, Israel; 5 Strauss Water Ltd, Or Yehuda, Israel; 6 Department of Preventive Medicine, School of Public Health, Faculty of Medicine, Tel Aviv University, Tel-Aviv, Israel; 7 Porter School of Environmental and Earth Sciences, Faculty of Exact Sciences, Tel Aviv University, Tel-Aviv, Israel; 8 Department of Statistics and Operations Research, Tel Aviv University, Tel-Aviv, Israel; 9 Sagol School of Neuroscience, Tel Aviv University, Tel-Aviv, Israel

**Keywords:** SARS-CoV-2, COVID-19, Long-COVID, Adulescens, Retrospective cohort study

## Abstract

**Background:**

The impact of long-term Coronavirus disease 2019 (COVID-19) on the pediatric population is still not well understood. This study was designed to estimate the magnitude of COVID-19 long-term morbidity 3–6 months after the date of diagnosis.

**Methods:**

A retrospective study of all Clalit Health Services members in Israel aged 1–16 years who tested positive for SARS-CoV-2 between April 1, 2020 and March 31, 2021. Controls, who had no previous diagnosis of COVID-19, were one-to-one matched to 65,548 COVID-19-positive children and teens, and were assigned the infection dates of their matches as their index date. Matching included age, sex, socio-economic score, and societal sector. Individuals were excluded from the study if they had severe medical conditions before the diagnosis such as cancer, diabetes, chronic respiratory diseases, and/or abnormal physiological development. Generalized Estimating Equations were used to estimate the associations between COVID-19 and the use of medical services. The analysis focused on the 3–6 months after the infection date. Adjustments were made for demographics and for the use of medical services 6–12 and 3–6 months before the infection date. The latter was necessary because of observed disparities in medical service utilization between the groups before the COVID-19 diagnosis, despite the matching process.

**Results:**

Statistically significant differences were only found for referrals for mental health services [adjusted relative-risk (RR) 1·51, 95%CI 1·15 − 1·96; adjusted risk-difference (RD) 0·001, 95%CI 0·0006 − 0·002], and medication prescriptions of any kind (RR 1·03, 95%CI 1·01–1·06; RD 0·01 95%CI 0·004 − 0·02).

**Conclusions:**

The significant increase in medication prescriptions and mental health service referrals support the hypothesis that COVID-19 is associated with long-lasting morbidities in children and adolescents aged 1–16 years. However, the risk difference in both instances was small, suggesting a minor impact on medical services.

**Supplementary Information:**

The online version contains supplementary material available at 10.1186/s13034-024-00736-w.

## Introduction

A substantial number of children and teens worldwide have been infected with severe acute respiratory syndrome coronavirus 2 (SARS-CoV-2), which causes coronavirus disease 2019 (COVID-19) [1, 2]. Children and teens are susceptible to SARS-CoV-2 infection but are frequently asymptomatic or paucisymptomatic [3]. In a small percentage of cases, complications such as pediatric inflammatory multisystem syndrome have been reported [4, 5]. Although there are detailed descriptions of the acute clinical course in children in the medical literature [3–5], few evidence-based studies have been published about the possible long-term morbidity of COVID-19 in the pediatric population, that is symptoms experienced after the acute phase of COVID-19 [6].

According to the World Health Organization [6], a Long- (or Post-) COVID condition is said to occur in individuals with a history of probable or confirmed SARS-CoV-2 infection, for a typical duration of 3 months after the onset of COVID-19. Long-COVID is considered to last for at least 2 months and cannot be explained by an alternative diagnosis. However, there are deviations in the definition of the time frame of Long-COVID [6]. Typical symptoms include fatigue, shortness of breath, and cognitive dysfunction that generally have an impact on everyday functioning [6]. In the adult population, there is growing evidence of Long-COVID morbidity. Antonelli et al. [7] found that 10·8% of all patients who tested positive for the Delta variant of SARS-CoV-2 virus experienced Long-COVID, and 4·5% of all patients infected with the Omicron variant experienced Long-COVID. Greenhalgh et al. [8] reported similar proportions, with approximately 10% of all patients experiencing Long-COVID. A subset of this population had serious sequalae that required intensive care but most Long-COVID patients reported mild symptoms such as cough, low grade fever, fatigue, and shortness of breath. In a prospective cohort study of 277 adults who recovered from COVID-19, Moreno-Perez et al. [9] detected symptoms of Long-COVID in approximately 50% of the cohort. The most commonly reported symptoms were fatigue, respiratory complaints, and neurological complaints. Carvalho-Schneider et al. [10] found that about two-thirds of all adults with non-critical COVID-19 were still experiencing some Long-COVID symptoms 60 days after the onset of the disease. Accordingly, Mendelson et al. [11] argued that health care systems should develop approaches to address the need for continued care for Long-COVID patients.

Evidence for Long-COVID in the pediatric population surfaced later than data for the adult population. The first type of documentation published were case reports [12–14], followed by small studies based on self or parental reports. Buonsenso et al. [15] examined a sample of 129 individuals aged 18 or younger, and reported that 20 of the 30 participants who were assessed 60–120 days after infection had persistent symptoms.

More recent studies of Long-COVID in children have used larger cohorts, but the findings are inconsistent. Molteni et al. [16] collected voluntary parental reports using a mobile application on 1734 children who tested positive for SARS-CoV-2, and their matched controls. They found that 4·4% of the recovering children had symptoms that lasted more than four weeks, whereas less than 1% of the matched controls were symptomatic for more than 28 days. Borch et al. [17] defined Long-COVID as symptoms lasting more than 4 weeks after diagnosis and found that 0·8% of the children in the sample self-reported Long-COVID symptoms on an electronic questionnaire. The most common symptoms reported in this study were fatigue, loss of smell, loss of taste, muscle weakness, dizziness, respiratory problems, and chest pain. Pinto Pereira et al. [18] administered questionnaires to compare 11–17 years olds, 6, and 12 months after their infection date and reported Long-COVID symptoms, especially tiredness, shortness of breath, poor quality of life, poor well-being, and fatigue. A meta-analysis of Long-COVID studies in children was conducted by Lopez-Leon et al. [19] The most prevalent clinical manifestations were mood changes (16·5%), fatigue (9·6%), and sleep disorders (8·4%). Racine et al. [20] conducted a meta-analysis on symptoms of depression and anxiety during the first year of the COVID-19 pandemic. Stephenson et al. [21] found that adolescents who were infected with COVID-19 were more likely to experience mental and physical symptoms 3 months after the infection date. In a national cross-sectional study, Kikenborg et al. [22] found a tendency towards better quality-of-life in the case group than in the controls, although more lasting symptoms were reported in the former.

Current studies of Long-COVID in pediatric populations tend to be based on small cohorts [12–16] or on self- or parental-reports [15–22], sometimes with a low response rate [23]. Not all studies include a control group [18–20, 24]. This makes it difficult to draw conclusions as to the profile or the prevalence of pediatric Long-COVID [24]. Since studies on the general population suggest that Long-COVID places a considerable burden on health services, that may require similar preparation in the eventuality of future pandemics [25], this study was designed to examine Long-COVID morbidity in a large pediatric population, based on objective clinical data, as compared to a matched control group. The findings can thus provide insights into the burden on service providers of Long-COVID in the pediatric population.

## Methods

### Study Population

Data were retrieved from Clalit Health Services (CHS), the largest health care provider in Israel, with 4·7 million members (53% of the population) from all sociodemographic groups of which the pediatric population accounts for 1·5 million members. The study was limited to CHS members aged 1–16 years who had been insured for at least 1 year prior to the beginning of the study on April 1, 2020. Individuals who had a confirmed diagnosis of diabetes, cancer, or developmental diseases (see Table S1 for list of diseases and codes) were excluded. The COVID-19 diagnosed group was composed of all CHS members who met the inclusion criteria and had a confirmed diagnosis of a SARS-CoV-2 infection using a polymerase chain reaction test from April 1, 2020, to March 31, 2021. The index date for each person in this group was the date of the first diagnosis of SARS-CoV-2 infection.

**Fig. 1:**
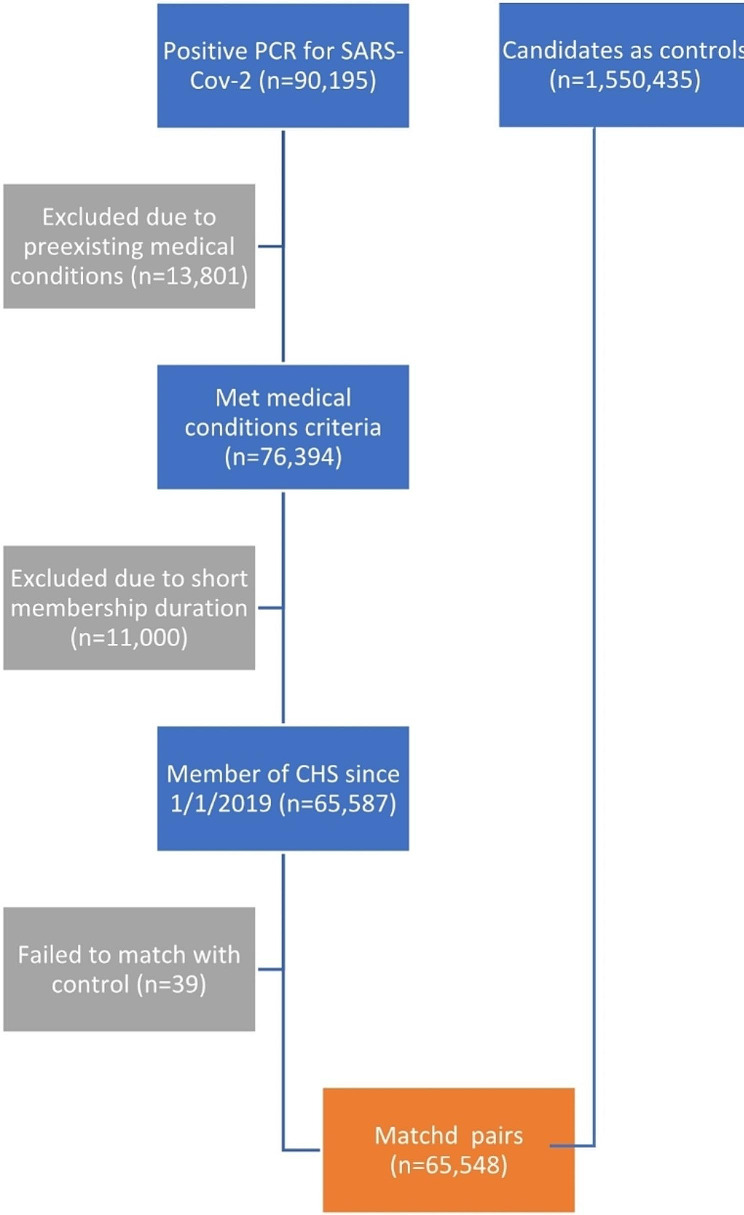
Consort diagram of the participant selection process

During this time frame, 90,195 participants under the age of 16 were diagnosed with SARS-CoV-2. Of these, 24,512 (27%) did not meet the inclusion criteria either due to pre-existing medical conditions (13,801, 15%) or because they had not been insured by CHS since April 1, 2019. Of these excluded participants the vast majority were diagnosed with lack of expected normal physiological development (ICD-10 R62 code) (11,633 cases, 13%). The prevalence of this diagnosis in the first year of life among CHS members is 14·8% which is similar, but not identical, to the rate observed in the participants of this study. This left 65,683 participants to match to the controls. The study excluded individuals over the age of 16 to avoid potential biases from medical evaluations related to Israeli army conscription. Figure 1 presents the selection process.

Each participant in the COVID-19 diagnosed group was matched without replacement with a single control CHS member who was not diagnosed with SARS-CoV-2 on or before the index date. Matching included month and year of birth, sex assigned at birth, socio-economic status (on a 3-point scale), societal sector (determined according to the registered address and divided into Jewish, Jewish Ultra-Orthodox, Arab, Bedouin, and ‘other’). Participants in the COVID-19 diagnosed group for which no match was found were excluded (135 cases, 0·2% of the cases). Each participant from the control group was assigned the same index date as the participant in the COVID-19 diagnosed group they were matched to. This left 65,548 participants in the COVID-19 diagnosed group and the same number of controls. The age distribution of the participants is presented in Figure S1 and the distribution of index date is presented in Figure S2.

### Study variables

Seven primary outcomes were defined within the 3–6 month period after the index date: (1) visits to primary physician, (2) visits to a specialty physician, (3) visits to the Emergency Room, (4) admission to a hospital, (5) prescriptions for any medication, (6) new diagnosis entered into the health record, and (7) referral for mental health services. Laboratory tests were initially considered as another possible outcome but were excluded since it was found that a large proportion of these tests were SARS-CoV-2 PCR tests and Israeli policy at the time only required these tests for people who had not previously tested positive for COVID-19.

**Fig. 2:**
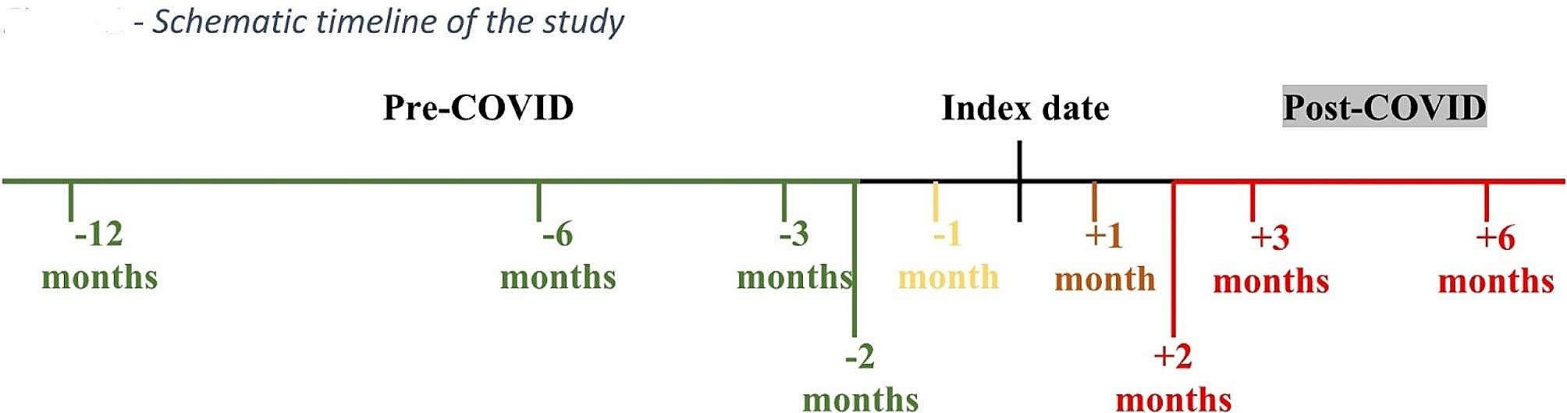
Schematic timeline of the study

The outcomes were recorded for the following periods: 6–12 months before the index date, 3–6 months before the index date, 2–3 months before and after the index date, 1–2 months before and after the index date, 2 weeks-1 month before and after the index date, 2 weeks before and 2 weeks after the index date (see Fig. 2 for a schematic timeline). The outcomes were converted to binary values indicating whether the service was used or not during the given period when used as dependent variables, which made it possible to quantify the relative risks of the outcomes in the infection vs. the control group. In addition to the matching variables and the outcomes, the data also included the total number of diagnoses registered in the Clalit Electronic Health Record (EHR) up to a year before the index date (Tables 1 and 2).

**Table 1:** Summary statistics of the categorial data used in this study

	Control group n (%)	COVID-19 diagnosed group n (%)	P-Value (${\varvec{\chi }}^{2}$)	Standardized Mean Difference (SMD)
**N**	65,548	65,548		
**Sex assigned at birth**			1·000	0
Male	33,042 (50·4)	33,042 (50·4)		
Female	32,506 (49·6)	32,506 (49·6)		
**3- point Socio-Economic Status**			< 0·001	0·046
Low	18,227 (27·8)	18,509 (28·2)		
Medium	38,657 (59·0)	38,588 (58·9)		
High	6223 (9·5)	5524 (8·43)		
No Data	2441 (3·7)	2927 (4·5)		
**Societal Sector**			1·000	< 0·001
Jewish	37,667 (57·5)	37,667 (57·5)		
Jewish Ultra-Orthodox	3820 (5·8)	3820 (5·8)		
Arab	13,244 (20·2)	13,244 (20·2)		
Bedouin	1703 (2·6)	1703 (2·6)		
Unknown / Other	9114 (13·9)	9114 (13·9)		
**District**			< 0·001	0·136
Dan – Petach-Tiqua	7111 (10·8)	8525 (13·0)		
Eilat	442 (0·7)	226 (0·3)		
South	9062 (13·8)	8311 (12·7)		
Haifa	9801 (15·0)	9438 (14·4)		
Jerusalem	8974 (13·7)	10,088 (15·4)		
Center	9594 (14·6)	10,215 (15·6)		
North	8568 (13·1)	7538 (11·5)		
Sharon – Shomron	8977 (13·7)	7858 (12·0)		
Tel-Aviv	2819 (4·3)	2862 (4·4)		
Missing	1 (0·0)	56 (0·1)		
Unknown	199 (0·3)	431 (0·7)		

**Table 2:** Summary statistics of the ordinal data used in this study

	Control group mean (SD)	COVID-19 diagnosed group mean (SD)	P-Value (Kruskal-Wallis)	Standardized Mean Difference (SMD)
**Age**	8·9 (4·0)	8·9 (4·0)	1·000	0
**Diagnoses in the EHR from birth to 12 months before index date**	0·86 (1·48)	0·87 (1·48)	< 0·001	0·011
**Use of medical services 6–12 month before index date**				
Primary Physician Visits	1·78 (2·37)	1·97 (2·48)	< 0·001	0·077
Specialty Physician Visits	0·40 (0·87)	0·42 (0·87)	< 0·001	0·028
ER Visits	0·06 (0·27)	0·07 (0·30)	< 0·001	0·024
Hospital Admissions	0·02 (0·20)	0·02 (0·17)	0·004	0·011
Lab Tests	5·79 (25·35)	6.38 (31·99)	< 0·001	0·020
Prescriptions	1·89 (3·55)	2·07 (3·76)	< 0·001	0·048
New Diagnoses	0·03 (0·22)	0·04 (0·21)	< 0·001	0·015
Referrals for mental health services	0·00 (0·08)	0·00 (0·07)	0·642	-0·003

### Study design

The study was a retrospective cohort study. The outcome variables for the infected and control groups differed 3–6 months before the index date, even when adjusting for available covariates. Therefore, the remaining analyses targeted the interaction between the infection and the period (before/after the index date) in the Generalized Estimating Equation (GEE) models with sandwich standard error estimators [26] applied to the data of each participant in the two periods. Correlations were assumed to be present between the measurements before/after the index date, as well as between each participant in the COVID-19 diagnosed group and the matched participant in the control group. The model was adjusted for outcomes 6–12 months before the index date, the calendar date of the index date dummy encoded at a resolution of 6 weeks beginning April 1, 2020, socio-economic status, sex assigned at birth, district, societal sector, and count of diagnoses registered in the EHR up to a year before the index date.

As negative controls, we applied the same model while using the period of 2–3 months before the index date and 1–2 months before the index date as the dependent variable. If the groups are comparable (there is no unmeasured confounding), an approximate null result was expected for the infection x period interaction term.

The G-formula was used to compute the marginal relative risks (RR) [26], while 95% confidence intervals and p-values were obtained using the bootstrap method with 999 bootstrap repetitions, and bootstrap sampling at the matched-pairs level. Holm’s method was used to adjust for multiple hypothesis testing for the primary outcomes of the study. GEE logistic regression models (Python statsmodels version 0·13·2) were used to adjust for confounders and account for inter-personal correlations [27]. To assess the sensitivity of the results to modeling assumptions, the results were compared to ones obtained by modified Generalized Additive Models (GAMs) and XGboost [28, 29].

## Results

### Study participants and matching

The descriptive statistics for the participants are presented in Tables 1 and 2. The main results are presented in Table 3. The large sample size makes even small differences statistically significant. Note however that the Standardized Mean Differences (SMDs) in Table 2 are smaller than 0.1, which is often considered to be the threshold for imbalance between two comparison groups. A small difference in socio-economic status remained despite matching on this variable, due to the delay between the time the matching was done (March 2022) and the time the data were retrieved (August 2022), during which a few changes occurred. Therefore, this variable was further adjusted for SES in the regression models. Figure S2 shows the distribution of the index date; i.e., the day when the infected participants were diagnosed with SARS-CoV-2. The index date distribution corresponds to the waves of the pandemic in Israel: the first wave in March-June 2020, the second wave in August-October 2020, and the third wave in December 2020-March 2021.

**Table 3:** Adjusted estimates for infection and period interactions

	Fraction in the Control Group (95%CI)	Fraction in the COVID-19 Diagnosed Group (95%CI)	Relative-Risk (95%CI)	Risk-Difference (95%CI)	Adjusted p-value
Primary Physician Visits	0·48 (0·47, 0·49)	0·48 (0·47, 0·48)	1·00 (0·98, 1·01)	-0·002 (-0·009, 0·004)	1
Specialty Physician Visits	0·17 (0·16, 0·17)	0·17 (0·17, 0·18)	1·03 (1·00, 1·07)	0·006 (0·0004, 0·01)	0·20
ER Visits	0·031 (0·028, 0·033	0·034 (0·032, 0·035)	1·10 (1·00, 1·20)	0·003 (0·00002, 0·006)	0·20
Hospital Admissions	0·008 (0·007, 0·009)	0·0077 (0·007, 0·0084)	1·02 (0·86, 1·21)	0·0001 (-0·001, 0·001)	1
Prescriptions	0·31 (0·30, 0·31)	0·32 (0·32, 0·32)	1·03 (1·01, 1·06)	0·01 (0·004, 0·02)	0·007
New Diagnoses	0·018 (0·016, 0·02)	0·016 (0·015, 0·017)	0·89 (0·79, 1·01)	-0·002 (-0·004, 0·0001)	0·23
Referrals for Mental Health Services	0·0028 (0·0022, 0·0036)	0·0042 (0·0037, 0·0047)	1·51 (1·15, 1·96)	0·001 (0·0006, 0·002)	0·018

The first step in the analysis was to ascertain that the COVID-19 diagnosed and control groups were exchangeable before the index date. For this purpose, the unadjusted RRs for the different outcomes were calculated **3–6 months before the index date** (Table 4). Despite the matching procedure, significant differences were observed between the COVID-19 diagnosed and the control groups before the index date, all of which indicated an increased risk in the COVID-19 diagnosed group: primary physician visits RR = 1·13, (95%CI 1·11 − 1·14), specialty physician visits RR = 1·10 (95%CI 1·07 − 1·12), lab tests RR = 1·23 (95%CI 1·2 − 1·26) and prescriptions RR = 1·11 (95%CI 1·09 − 1·13). Table 4 shows that adjusting for the values of the outcomes 6–12 months before the index date, age, calendar date (of the index date), socio-economic status, sex assigned at birth, societal sector, district, and the number of registered diseases in the medical record 12 months before the index date did not alleviate the problem.

**Table 4:** Negative control: outcomes compared 6 − 3 months before index date

	Control group proportion	COVID-19 diagnosed group proportion	Unadjusted RR (CI)	Adjusted OR (CI)
Use of medical services 3–6 month before index date				
Primary Physician Visits	0·38	0·43	1·09 (1·08, 1·10)	1·13 (1·11,1·14)
Specialty Physician Visits	0·14	0·15	1·07 (1·04, 1·10)	1·1 (1·07,1·12)
ER Visits	0·02	0·03	1·05 (0·98, 1·12)	1·07 (1,1·14)
Hospital Admissions	0·01	0·01	1·08 (0·95, 1·22)	1·13 (1,1·28)
Lab Tests	0·16	0·19	1·20 (1·17, 1·23)	1·23 (1·2,1·26)
Prescriptions	0·24	0·27	1·06 (1·05, 1·08)	1·11 (1·09,1·13)
New Diagnoses	0·01	0·01	0·96 (0·87, 1·06)	0·99 (0·9,1·1)
Referrals for Mental Health Services	0·00	0·00	0·84 (0·66, 1·04)	0·85 (0·67,1·08)

Figure S3 presents the unadjusted RRs and the 95% CIs of SARS-CoV-2 infection for using different health services in different time windows, defined relative to the index date. The figure depicts the progression from the viewpoint of the health service providers, in particular during the acute phase of the disease. An increase in the use of lab tests and visits to primary and secondary physicians was followed by an increase in new diagnoses and ER visits, and subsequently by hospital admissions and prescriptions. Figure S3 also shows that there was a difference between the COVID-19 diagnosed group and the control group even 6–12 months before the index date so that the matching did not fully adjust for the differences between the populations.

### Relative risks and risk differences

To further adjust for the differences between the control and COVID-19 diagnosed groups, the outcomes were modelled while controlling for participants’ variables using GEE logistic regression [26]. Two different periods were included for both the COVID-19 diagnosed group and the control group. In this model, the RRs of interest are expressed as the interaction term between the infection and period (see Methods).

The relative risks between the COVID-19 diagnosed group and the control group and risk differences are presented in Table 3 with the aforementioned adjustments to the period 3–6 months before the index date. The results revealed statistically significant differences in two outcomes: prescriptions (RR 1·03, 95%CI 1·01–1·06, adjusted *P* = .007), and referrals for mental health services(RR 1·51, 95%CI 1·15 − 1·96, adjusted *P* = .018).

As a negative control analysis, the analysis was repeated while replacing the outcome periods by the periods of 2–3 and 1–2 months **before** the index date; i.e., by shifting the index date to 2 months before the diagnosis of infection. The results showed that the differences between the COVID-19 diagnosed group and the control group were negligible although statistically significant (Table 5). Since the periods are short (1 month) for rare outcomes not all bootstrap rounds converged.

**Table 5:** Negative control for parallel trends - comparing the periods 3 − 2 months and 2 − 1 months before infection

	Proportion in COVID-19 diagnosed group 3 − 2 (2 − 1) month before index date	Proportion in control group 3 − 2 (2 − 1) month before index date	p-value	Relative-Risk (CI)
Primary Physician Visits	0·202 (0·202)	0·179 (0·180)	0·04	0·98 (0·96, 1·00)
Specialty Physician Visits	0·064 (0·066)	0·057 (0·059)	0·26	1·02 (0·98, 1·07)
ER Visits	0·009 (0·009)	0·008 (0·008)	0·91	1·01 (0·90, 1·13)
Hospital Admissions	0·003 (0·003)	0·002 (0·003)	0·77	1·04 (0·85, 1·29)
Prescriptions	0·123 (0·123)	0·108 (0·110)	0·03	0·97 (0·94, 1·00)
New Diagnoses	0·005 (0·005)	0·004 (0·005)	0·48	1·06 (0·91, 1·24)
Referrals for Mental Health Services	0·001 (0·001)	0·001 (0·001)	0·40	0·87 (0·59, 1·22)

Sensitivity to modeling assumption of linear relation between independent variables and the log odds of the dependent variables was assessed using GAMs (Table S2) and XGboost (Table S3) [28, 29]. In both cases, the results for all outcomes other than referrals for mental health services were not statistically significant. For mental health services, the results were similar to the results obtained using logistic regression models, although the p-value when using GAMs exceeded 0·05 (GAMs: 1·26 95%CI 1·05 − 1·55, adjusted p-value = 0·08; XGboost: 1·21 95%CI 1·05 − 1·39 adjusted *P* = 0·03). The interpretable GAM results for the different outcomes are provided in Figures S4–S10.

## Discussion

This study investigated the associations between SARS-CoV-2 pediatric infections and recourse to health services 3–6 months after the infection, while controlling for socio-economic status, existing medical conditions, existing health service consumption, age, and date of diagnosis. Of the seven health services examined, the only statistically significant differences were for referrals for mental health services (RR 1·51 95%CI 1·15 − 1·96), and prescriptions (RR 1·03 95%CI 1·01–1·06), thus confirming the association between COVID-19 and long-term morbidity. Although the relative risk was high for referrals for mental health services, the rates of referrals in this age group were low, as was the risk difference (adjusted RD 0·001 95%CI 0·0006 − 0·002). Similarly, the risk difference for prescriptions was also small (adjusted RD 0·01 95%CI 0·004 − 0·02). There were no other statistically significant associations between the infection and other health services. This includes primary physician visits (RR 1·00 95%CI 0·98 − 1·01), specialty physician visits (RR 1·03 95%CI 1·00–1·07), ER visits (RR 1·1 95%CI 1·00–1·20), hospital admissions (RR 1·02 95%CI 0·86 − 1·21), and new diagnoses (RR 0·89 95%CI 0·79 − 1·01). However, some of these services are rarely used by children so that no definitive conclusions can be drawn despite the relatively large cohort in this study (*N* = 65,548 for the COVID-19 diagnosed group and for the control group). Hence, the evidence suggests that the impact of pediatric long- COVID on the health care system for this cohort was small.

The mechanism behind the increase in referrals for mental health services is not clear and can be attributed to either physiological or psychological reasons, or both. Another possible explanation may be related to the quarantine period (14 days), which was mandatory in Israel at the time for individuals with COVID-19. However, the quarantine in itself cannot explain the difference in referrals for mental health services because at the time of the study, anyone exposed to COVID-19 was required to quarantine, except individuals who had already had COVID-19. Note that the pediatric population of in the control group underwent more lab tests than the COVID-19 diagnosed group, both during the 3–6 months, and 1–3 months after the index date (see Figure S3). Hence, the control group appears to have had a higher frequency of PCR tests as a result of exposure to individuals with COVID-19, thus leading to potentially higher frequencies of quarantine periods compared to quarantines in the COVID-19 diagnosed group.

One of the major strengths of this study was the use of nationwide data and objective outcome measures, unlike most other studies that have either been small in size [12–16] or relied on self or parental reports [15–22]. The large, detailed dataset made it possible to match the COVID-19 diagnosed group to a control group and adjust for additional confounders.

However, this study is not without limitations. Referrals to medical services are indirect measures of morbidity. In addition, data regarding the duration of the symptoms and the time to resolution of the medical complaints were unavailable. Further, some symptoms, such as fatigue, might not manifest in changes in the use of medical services patterns. Another possible limitation is that the COVID-19 diagnosed group was composed of children and teens who tested positive and did not necessarily include all the infected children at the time of the study. Nonetheless, during the first 3 waves of COVID-19 in Israel, testing was rather stringent, and infections would have been identified well [30]. Note that despite the adjustments for past use of medical services, sex assigned at birth, age, date, societal sector, socio-economic status, and district, significant differences still remained between the COVID-19 diagnosed group and the control group 3–6 months prior to the index date. This was accounted for statistically in the main outcomes of this study by adjusting for these differences; however, other unmeasured risk factors may have been present.

Our analysis included controls who did not test positive before the index date. However, some controls tested positive for COVID-19 during the 6 month of post index-date follow-up period. To test the influence of these cases, we repeated the analyses while excluding the 2,348 matched pairs (3·58%) where controls tested positive in this timeframe. The reproduced results in Table S4 showed very high congruence with our main results, affirming that our findings are robust with respect to the design choice.

## Conclusion

This study supports previous evidence pointing to the existence of pediatric Long-COVID, by drawing for the first time on a large cohort and objective health care outcomes. Our study revealed that individuals who later tested positive for COVID-19 were already accessing healthcare services more frequently than controls months before their diagnosis, suggesting previously unidentified risk factors. It also points to specific medical resources, especially mental health services, that might experience an increase in demand. However, the findings also suggest that the extent of the increase in demand for health services was small.

### Electronic supplementary material

Below is the link to the electronic supplementary material.

Supplementary Material 1

Supplementary Material 2

Supplementary Material 3

Supplementary Material 4

Supplementary Material 5

Supplementary Material 6

Supplementary Material 7

Supplementary Material 8

## Data Availability

Data used to generate the results reported in this study will be made available following publication to researchers who provide a methodologically sound proposal. Data will only be made available if approval is granted from the Clalit Health Services Ethics Committee and a data transfer agreement is signed. Requests should be directed to the corresponding author.
