# Supplementary figures and images for: Impact of Long-COVID in children: a large cohort study
Source: Child Adolesc Psychiatry Ment Health. 2024 Apr 15;18:48. doi: 10.1186/s13034-024-00736-w (PMC11020876; doi:10.1186/s13034-024-00736-w)

Figure S4 - GAM for predicting primary physician visits.


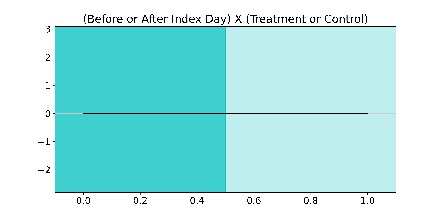

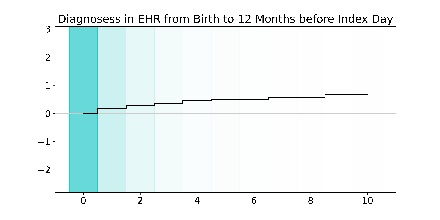

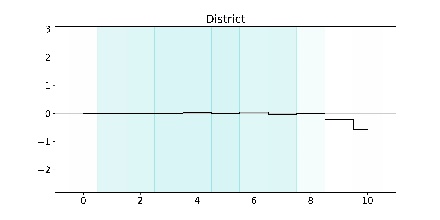

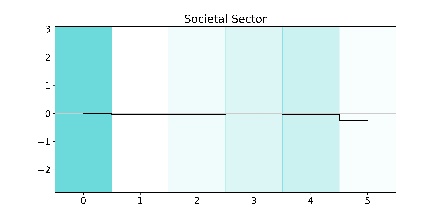

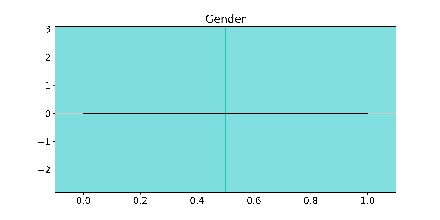

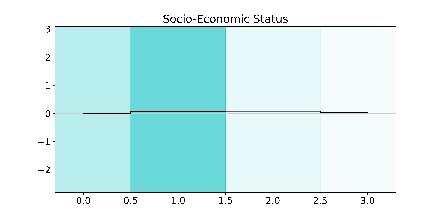

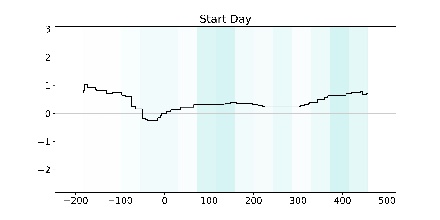

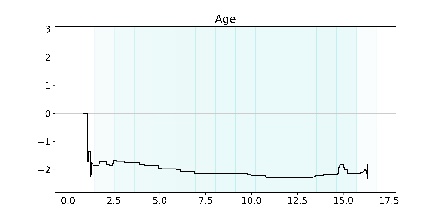

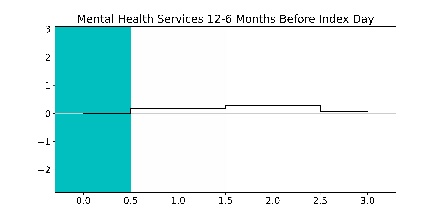

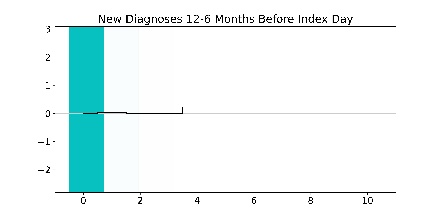

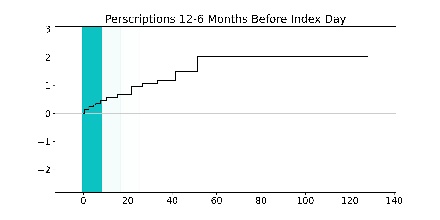

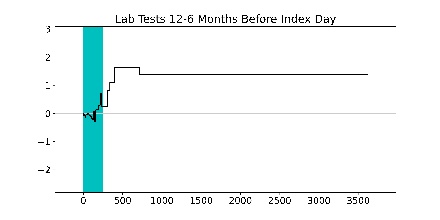

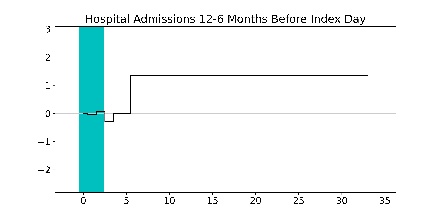

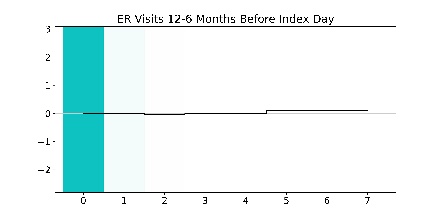

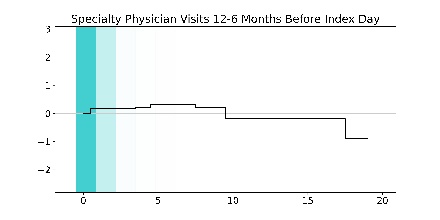

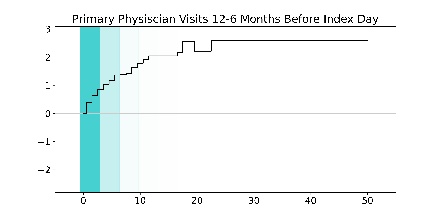

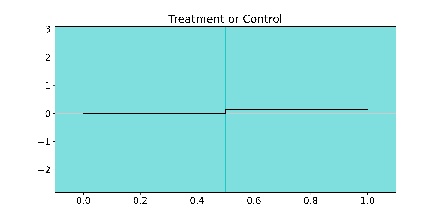

Supplement: Supplementary file 1 — Supplementary Material 1 [file 13034_2024_736_MOESM1_ESM.docx]

Figure S5 - GAM for predicting consulting physician visits.


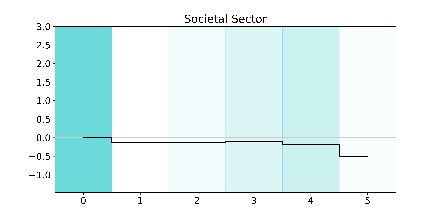

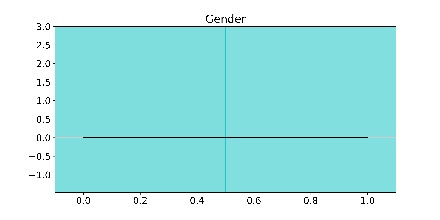

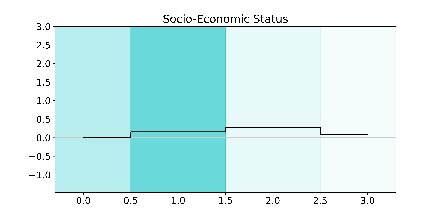

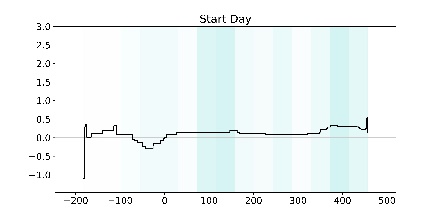

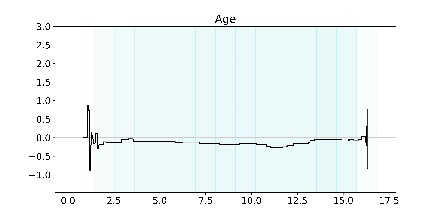

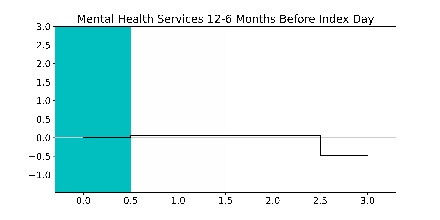

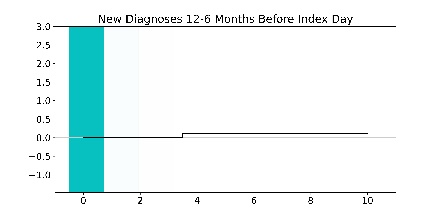

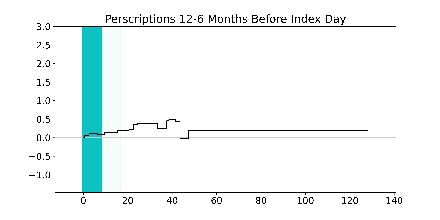

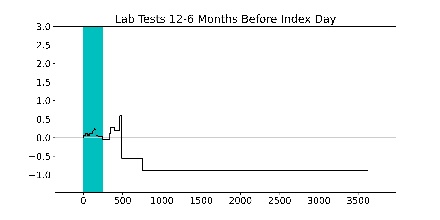

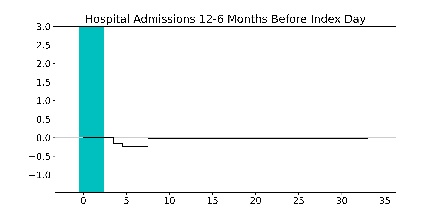

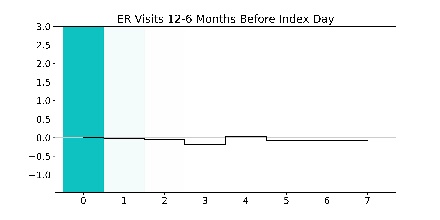

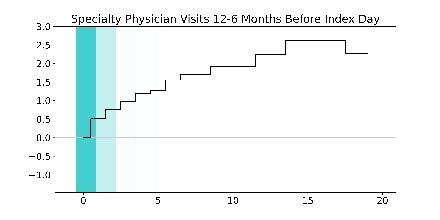

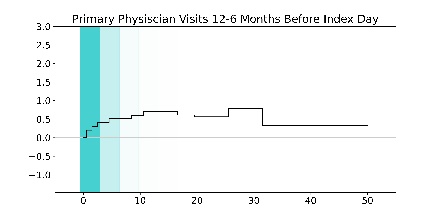

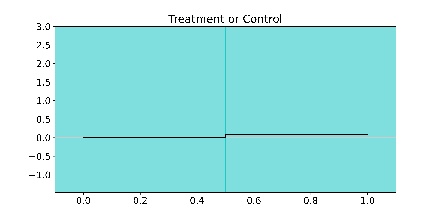

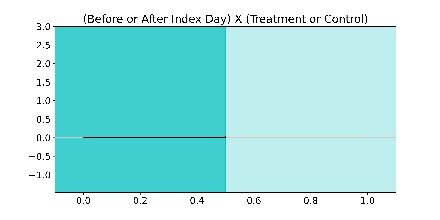

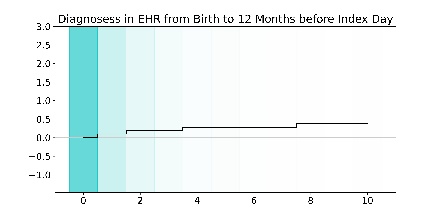

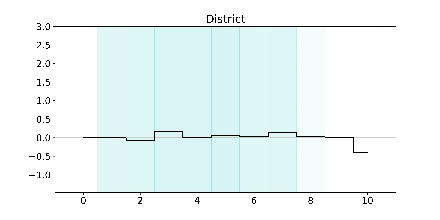

Supplement: Supplementary file 2 — Supplementary Material 2 [file 13034_2024_736_MOESM2_ESM.docx]

Figure S7 - GAM for predicting hospital admissions.


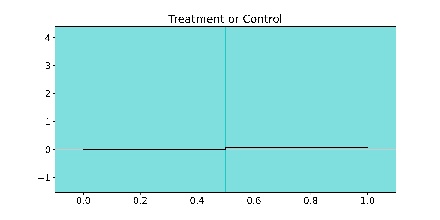

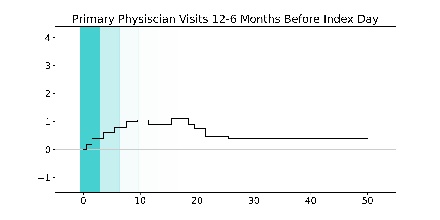

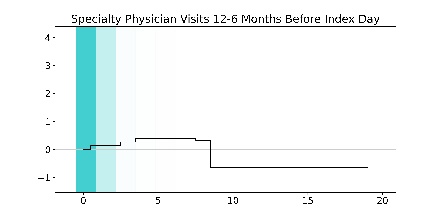

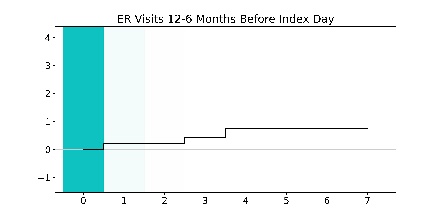

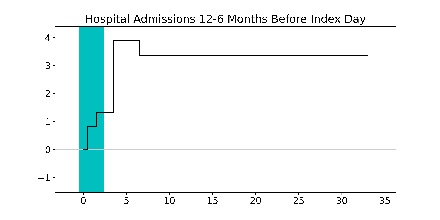

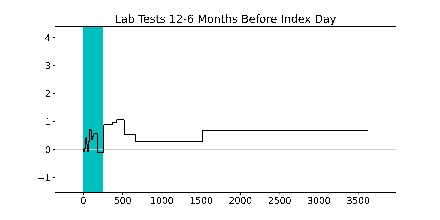

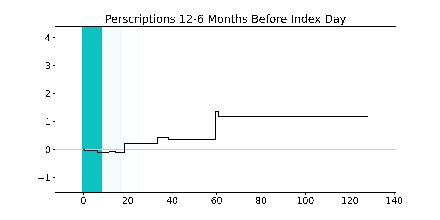

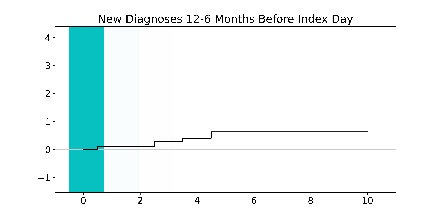

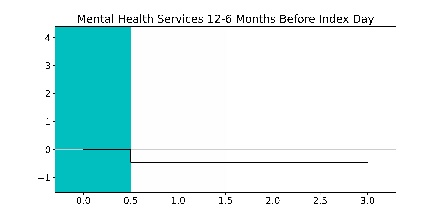

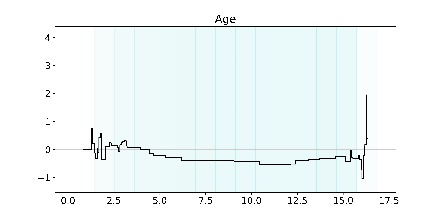

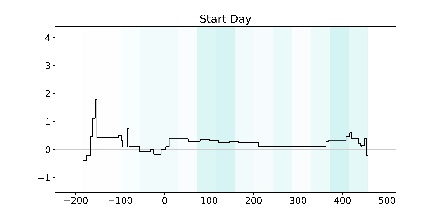

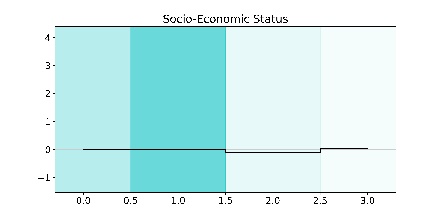

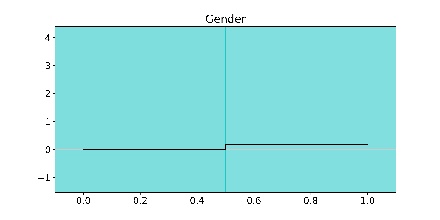

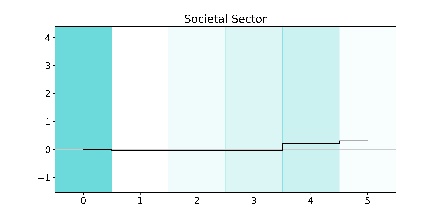

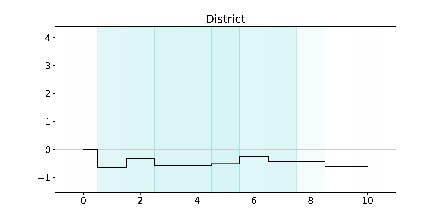

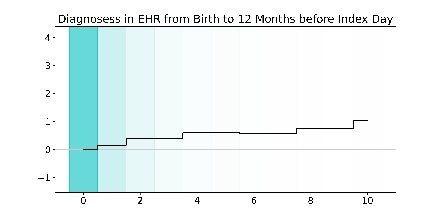

Supplement: Supplementary file 3 — Supplementary Material 3 [file 13034_2024_736_MOESM3_ESM.docx]

Figure S8 - GAM for predicting medication prescriptions.


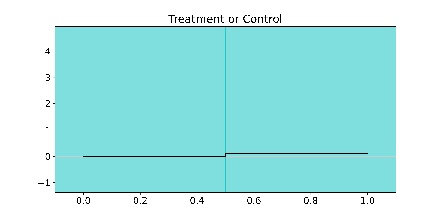

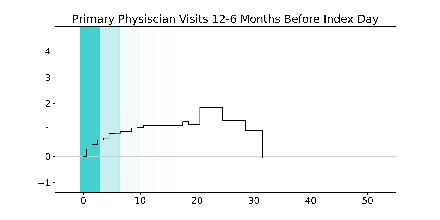

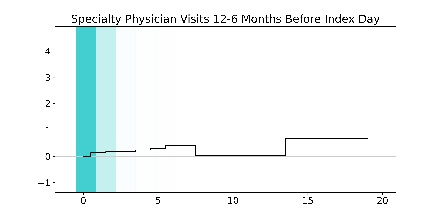

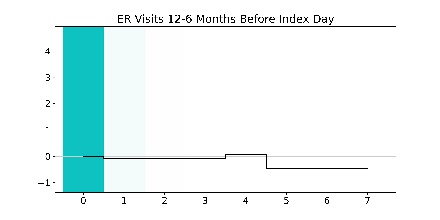

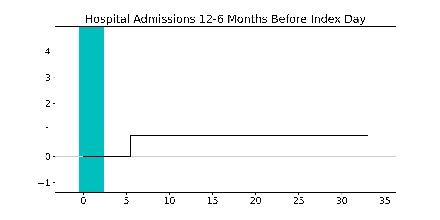

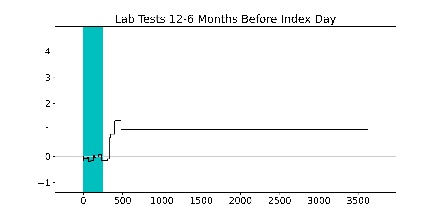

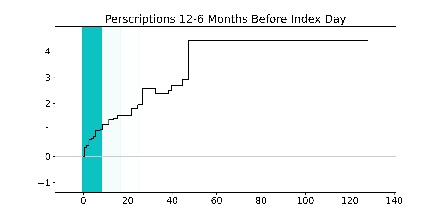

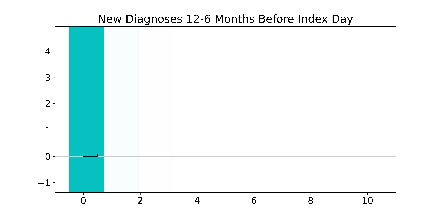

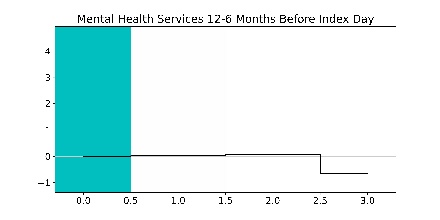

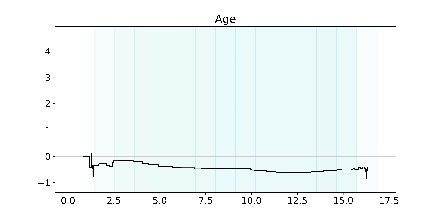

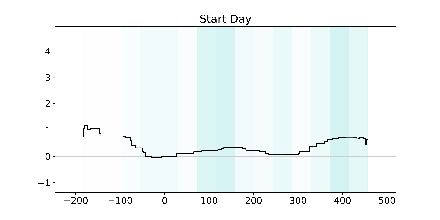

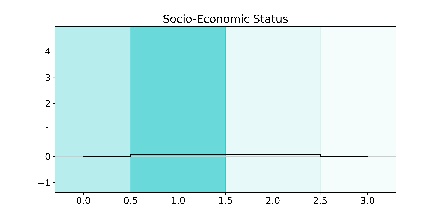

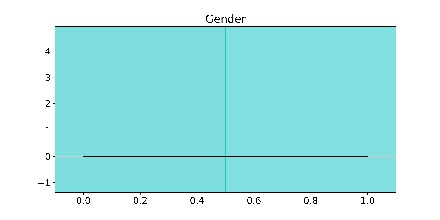

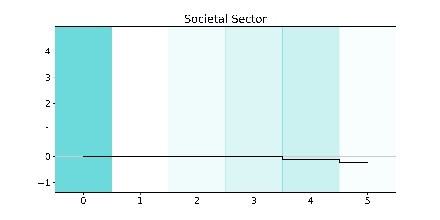

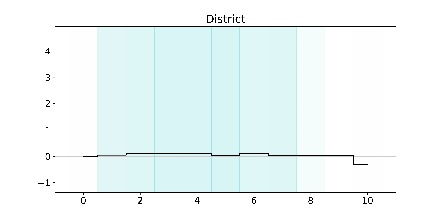

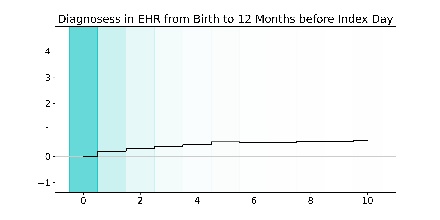

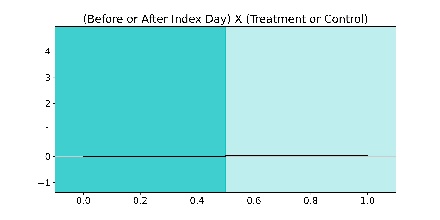

Supplement: Supplementary file 4 — Supplementary Material 4 [file 13034_2024_736_MOESM4_ESM.docx]

Figure S9 - GAM for predicting new registered problems in EHR.


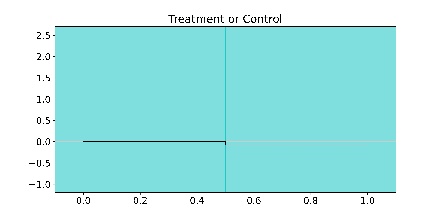

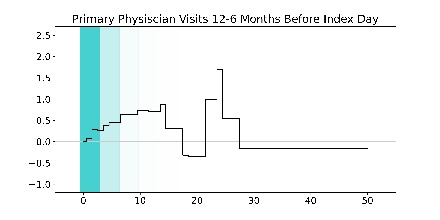

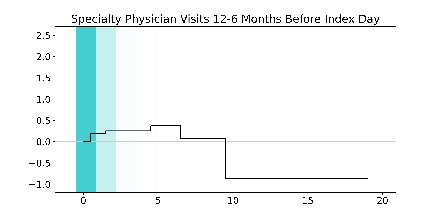

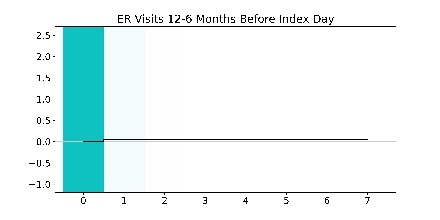

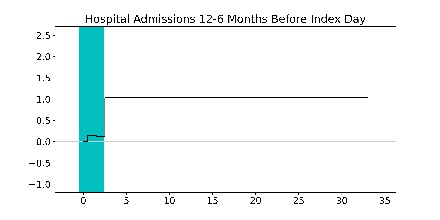

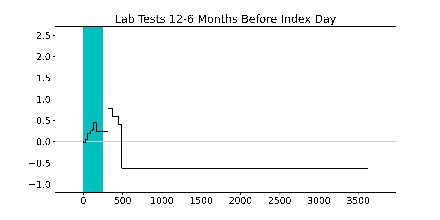

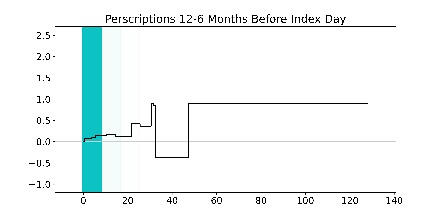

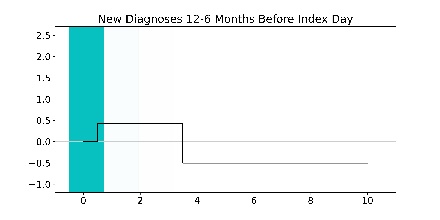

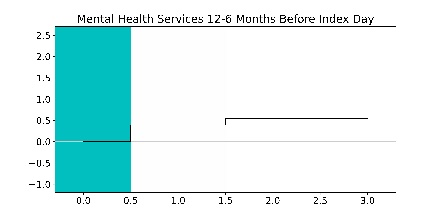

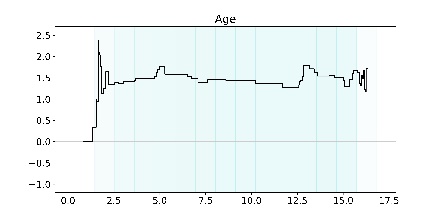

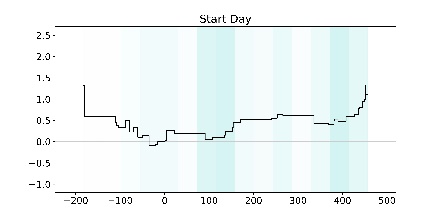

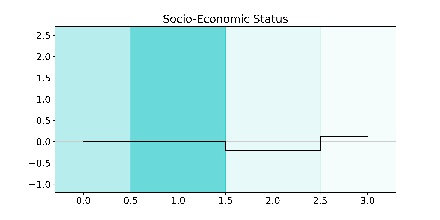

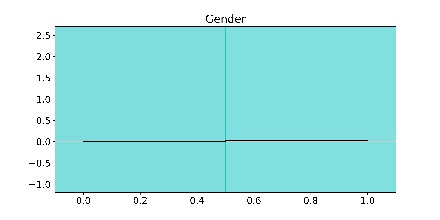

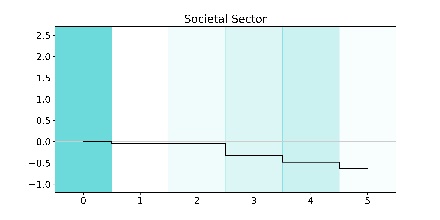

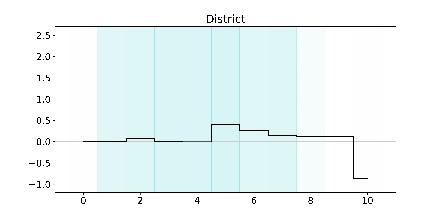

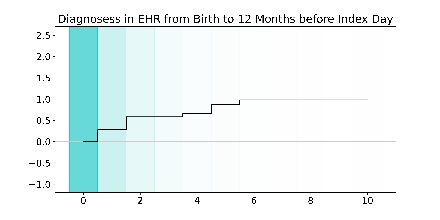

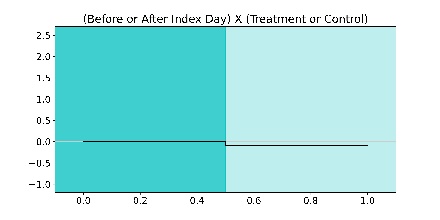

Supplement: Supplementary file 5 — Supplementary Material 5 [file 13034_2024_736_MOESM5_ESM.docx]

Figure S10 - GAM for predicting emotional treatment.


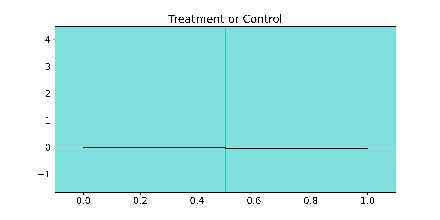

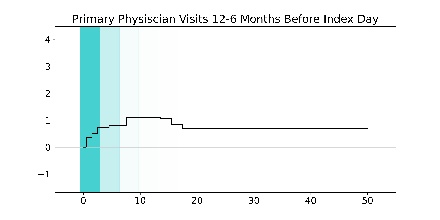

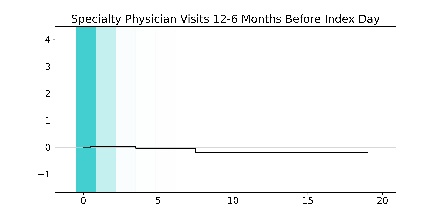

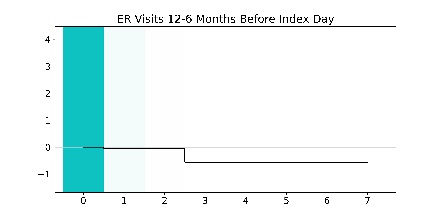

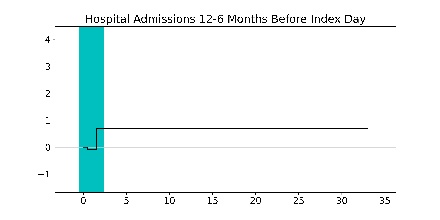

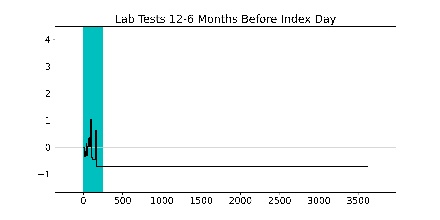

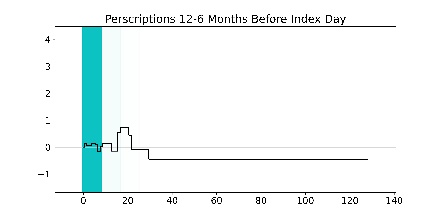

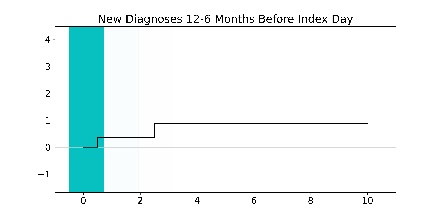

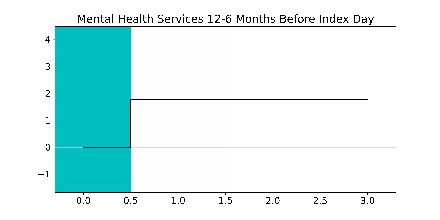

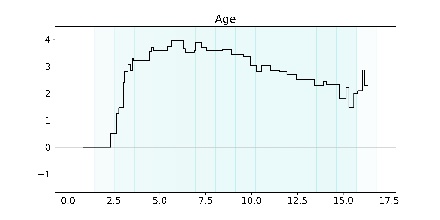

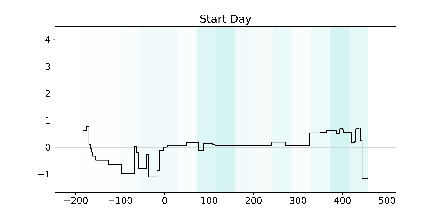

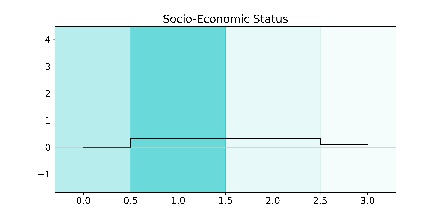

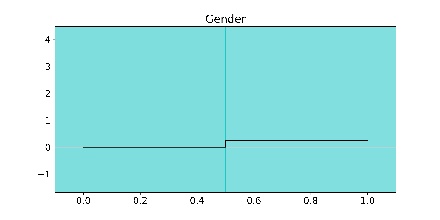

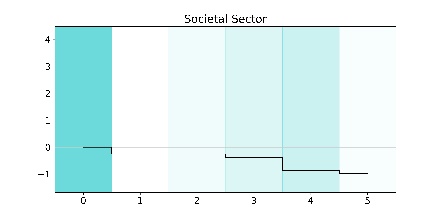

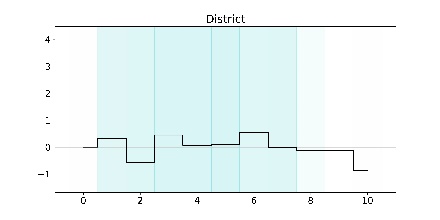

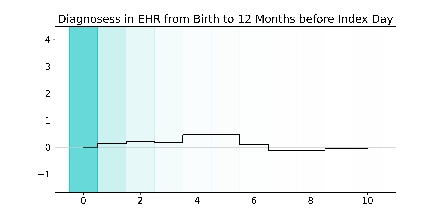

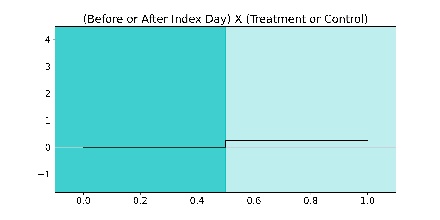

Supplement: Supplementary file 6 — Supplementary Material 6 [file 13034_2024_736_MOESM6_ESM.docx]

Figure S6 - GAM for predicting ER visits.


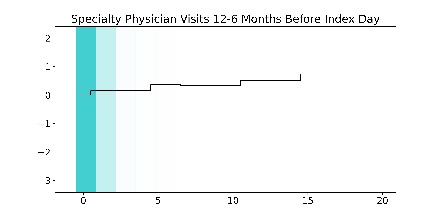

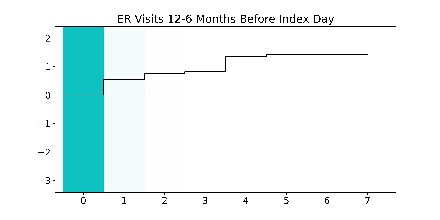

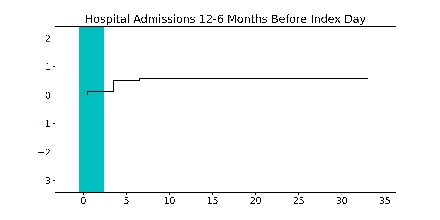

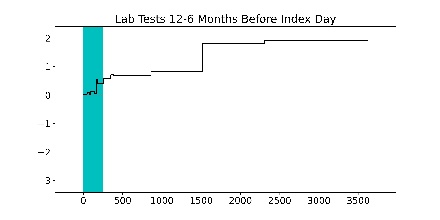

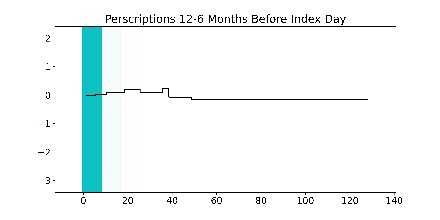

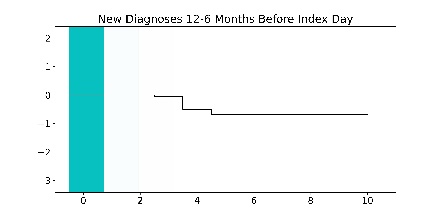

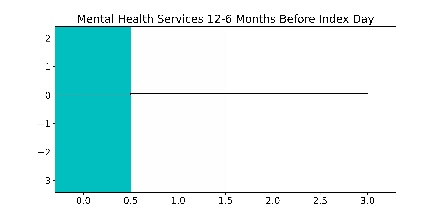

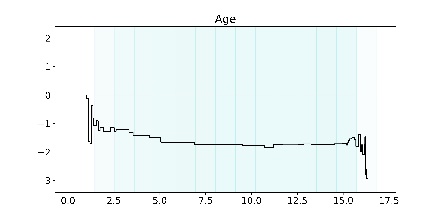

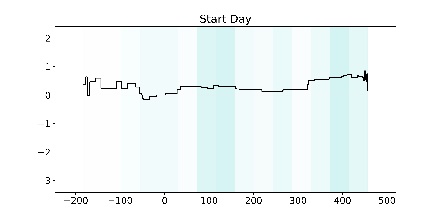

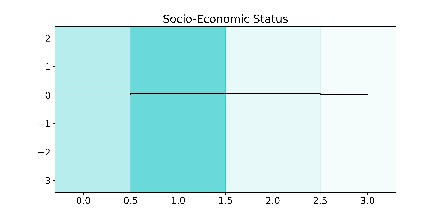

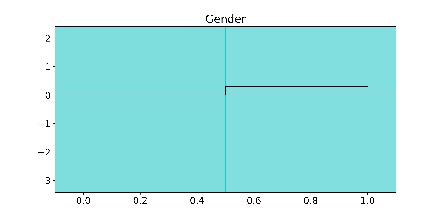

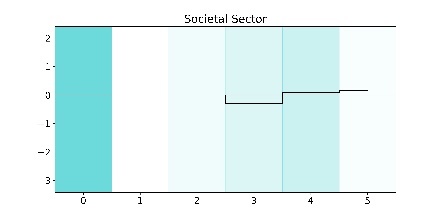

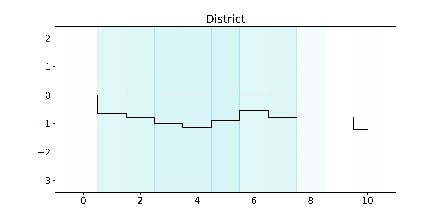

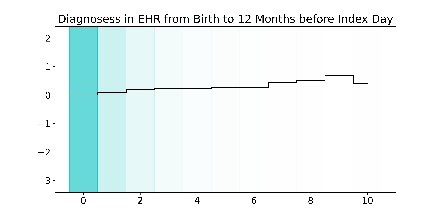

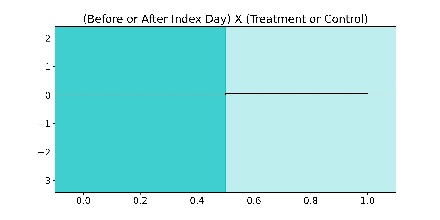

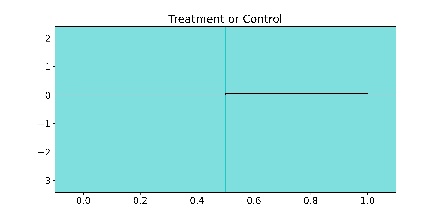

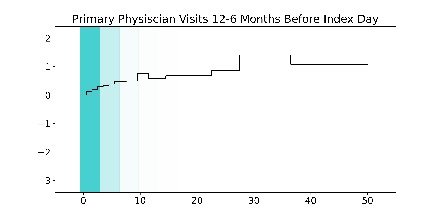

Supplement: Supplementary file 7 — Supplementary Material 7 [file 13034_2024_736_MOESM7_ESM.docx]
