# Supplementary material for: Impact of Long-COVID in children: a large cohort study
Source: Child Adolesc Psychiatry Ment Health. 2024 Apr 15;18:48. doi: 10.1186/s13034-024-00736-w (PMC11020876; doi:10.1186/s13034-024-00736-w)
Supplement: Supplementary file 8 — Supplementary Material 8 [file 13034_2024_736_MOESM8_ESM.docx]

###### **Supplementary material**

**A.1 List of Figures and Tables in the online only material**

Table S1 - Diagnostic codes that were used to exclude participants from the study.

Table S2 - The estimated effect of Covid-SARS-2 using GAMs trained on the period 3-6 month before covid to the period 3-6 month after.

Table S3 - The estimated effect of Covid-SARS-2 using Extreme Gradient Boosting trained on the period 3-6 month before covid to the period 3-6 month after.

Figure S1 - Age of study participants at the index date

Figure S2 - Distribution of the index date, that is, the day the disease was acquired.

Figure S3 - Unadjusted relative risk for use of different services before and after the index date.

Figure S4 - GAM for predicting primary physician visits.

Figure S5 - GAM for predicting consulting physician visits.

Figure S6 - GAM for predicting ER visits.

Figure S7 - GAM for predicting hospital admissions.

Figure S8 - GAM for predicting medication prescriptions.

Figure S9 - GAM for predicting new registered problems in EHR.

Figure S10 - GAM for predicting emotional treatment.

## **A.2 Robustness**

To verify that modeling assumptions and data discretization are not responsible for the results obtained we trained Generalized Additive Models (GAMs)^1^ by training extreme gradient boosting models (XGBoost)^2^ model on stumps. By using this method, we can avoid data discretization and normalization. This also allows us to present the model in an interpretable way that allows seeing the influence of the different attributes on the predicted outcome. The model was trained to predict each outcome using logit as a link function and log loss. We note that it has been shown that both the log-loss and the use of small trees allow boosted models to generate calibrated models.^3^ The results of these models are presented in Section E.2.

To verify that feature interactions are not responsible for the results obtained we trained extreme gradient boosting models (XGboost) where trees could use any combination of features with the only restriction being that the feature representing the interaction with having the covid disease and the measured period being after the index date can only be used as a separate tree. This means that the generated model has the structure $\sigma\left( w_{0}+w_{1}\text{covid}X\text{after}+g\left( \text{all other features} \right) \right)$ where the function $g$ is an ensemble of trees. Therefore, it is possible to measure the log-odds-ratio predicted by this model as well as the relative-risk by using the G-formula. The results are presented in Table S3.

The results obtained using GAMs (Table S2) and the results obtained using XGboost models (Table S3) are very similar to the results obtained using Generalized Estimating Equations (Table 2). Therefore, we conclude that the results are robust to modeling assumptions such as linearity and small feature interaction, and robust to data discretization and normalization.

**A.3 Explainability of models**

GEE is the main model used in this study. The assumption in the GEE model is of linear relation between the log-odds and the independent variables (features). I.e., the GEE model assumes that

$$log-odds=\theta+\sum w_{i}x_{i}$$

where θ is a scalar, $x_{i}$ is the $i$’th feature, and $w_{i}$ is a weight associated with it. In the GAMs model that we used; the assumption is that:

$$log-odds=\sum f_{i} (x_{i} )$$

where $f_{i}$ can be any function that the XGBoost algorithm learns from the data, not restricted to being just a scalar multiplier of the feature $x_{i}$. As described in Section A.1 GAMs generate explainable models. Figure S‎4 – Figure S10 present the GAMs for the different outcomes. Each figure shows the contribution of the different features where for each feature the graph can be interpreted as estimates of the log-odds-ratio associated with the different value of the feature compared to the value zero. The background color presents the density at the different levels of the features. Features that do not have any influence on the outcome according to the model are not presented.

A.4 COVID-19 cases from the control group

The study design, although retrospective, tries to mimic RCTs in the sense that treatments and controls are being selected in the index date without using information that will only be determined in the future. To verify that this does not create a bias towards the null hypothesis, we repeated the analysis while removing matched pairs if the control tested positive for COVID-19 during the 6 months after the index date, since the main outcomes were measured 3-6 month after the index date. Out of the 65548 controls in the study, 2348 cases (3.58%) tested positive for COVID-19 during the 6 month follow up period after the index date. We used the same bootstrapping and modeling technique to generate the equivalent of Table 3 when removing these pairs. The results are qualitatively similar to the results of Table 3 and are provided in Table S4

|  |  |  |  |  |  |
| --- | --- | --- | --- | --- | --- |
|  |  |  |  |  |  |
|  |  |  |  |  |  |
|  |  |  |  |  |  |
|  |  |  |  |  |  |
|  |  |  |  |  |  |
|  |  |  |  |  |  |
|  |  |  |  |  |  |

## **References**

1. Hastie, TJ., Tibshirani R. Generalized additive models. Statistical models in S. Routledge, 2017: 249-307.

2. Chen, T., Guestrin, C.. Xgboost: A scalable tree boosting system. In Proceedings of the 22nd ACM SIGKDD international conference on knowledge discovery and data mining. 2016: 785-794.

3. Niculescu-Mizil, A., Caruana, R. (2005, July). Obtaining Calibrated Probabilities from Boosting. In UAI, 2005;**5**: 413-20.

Table S1 - Diagnostic codes that were used to exclude participants from the study.

| **ICD-10 code** | **Description** | **Number of cases** |
| --- | --- | --- |
| C00 | Malignant neoplasm of lip | 8 |
| C44 | Other malignant neoplasms of skin | 7 |
| C95 | Leukemia of unspecified cell type | 7 |
| E10 | Type 1 diabetes mellitus | 61 |
| E11 | Non-insulin-dependent diabetes mellitus | 7 |
| E13 | Other specified diabetes mellitus | 131 |
| E84 | Cystic fibrosis | 16 |
| F84 | Autistic disorder | 690 |
| G40 | Epilepsy and recurrent seizures | 590 |
| G80 | Cerebral palsy | 95 |
| P27 | Chronic respiratory disease originating in the perinatal period | 74 |
| Q90 | Down syndrome | 95 |
| Q99 | Other chromosome abnormalities, not elsewhere classified. | 7 |
| R62 | Lack of expected normal physiological development | 11719 |
| R62·50 | Unspecified lack of expected normal physiological development in childhood | 8905 |
| R62·52 | Short stature (child) | 2320 |
| R62·51 | Failure to thrive (child) | 408 |
| R62·0 | Delayed milestone in childhood | 86 |
| N07 (AMB) | Hereditary nephropathy, not elsewhere classified | 223 |
| 7707 (ATD) | Chronic respiratory disease arising in the perinatal period | 25 |
| 7580 (ATD) | Down's syndrome | 13 |
| 3456 (AMB) | Infantile spasms | 4 |
| 3455 (AMB) | Localization-related (focal) (partial) epilepsy and epileptic syndromes with simple partial seizures | 3 |
| 2504 (AMB) | Diabetes with renal manifestations | 2 |
| 2000 (AMB) | Reticulosarcoma | 2 |
| 3454 (AMB) | Partial epilepsy, with impairment of consciousness | 1 |

Table S2 - The estimated effect of Covid-SARS-2 using GAMs trained on the period 3-6 month before covid to the period 3-6 month after.

| **Outcome** | **Proportion in infected (Covid) group 6-3 month before (3-6 month after) index date** | **Proportion in control group 6-3 month before (3-6 month after) index date** | **p-value (corrected for multiple hypothesis testing)** | **Relative-Risk (CI)** | **Converged bootstrap rounds (of 999)** |
| --- | --- | --- | --- | --- | --- |
| Primary Physician Visits | 0·428 (0·484) | 0·380 (0·484) | 1·0 | 1·00 (0·99, 1·00) | 999 |
| Consulting Physician Visits | 0·152 (0·176) | 0·138 (0·156) | 0·4 | 1·03 (1·00, 1·05) | 999 |
| ER Visits | 0·025 (0·034) | 0·024 (0·029) | 0·51 | 1·04 (1·00, 1·13) | 999 |
| Hospital Admissions | 0·008 (0·008) | 0·007 (0·007) | 1·0 | 1·01 (0·95, 1·11) | 999 |
| Medication Prescriptions | 0·268 (0·324) | 0·243 (0·284) | 0·21 | 1·02 (1·00, 1·03) | 999 |
| New Diagnoses | 0·012 (0·016) | 0·012 (0·019) | 0·21 | 0·92 (0·83, 1·00) | 999 |
| Emotional Treatments | 0·002 (0·004) | 0·002 (0·003) | 0·08 | 1·26 (1·05, 1·55) | 999 |

Table S3 The estimated effect of Covid-SARS-2 using Extreme Gradient Boosting trained on the period 3-6 month before covid to the period 3-6 month after.

| **Outcome** | **Proportion in infected (Covid) group 6-3 month before (3-6 month after) index date** | **Proportion in control group 6-3 month before (3-6 month after) index date** | **p-value (corrected for multiple hypothesis testing)** | **Relative-Risk (CI)** | **Converged bootstrap rounds (of 999)** |
| --- | --- | --- | --- | --- | --- |
| Primary Physician Visits | 0·428 (0·484) | 0·380 (0·484) | 1·0 | 1·00 (0·99, 1·00) | 249 |
| Consulting Physician Visits | 0·152 (0·176) | 0·138 (0·156) | 1·0 | 1·02 (1·00, 1·05) | 249 |
| ER Visits | 0·025 (0·034) | 0·024 (0·029) | 1·0 | 1·05 (1·00, 1·12) | 249 |
| Hospital Admissions | 0·008 (0·008) | 0·007 (0·007) | 1·0 | 1·02 (0·99, 1·10) | 249 |
| Medication Prescriptions | 0·268 (0·324) | 0·243 (0·284) | 0·27 | 1·02 (1·00, 1·04) | 249 |
| New Diagnoses | 0·012 (0·016) | 0·012 (0·019) | 0·34 | 0·93 (0·85, 1·00) | 249 |
| Emotional Treatments | 0·002 (0·004) | 0·002 (0·003) | 0·03 | 1·21 (1·05, 1·39) | 249 |

Table S4 Reproduction of the main results when removing matched pairs in which the control acquired COVID-19

|  | **P0 (95%CI)** | **P1 (95%CI)** | **Relative-Risk (95%CI)** | **Risk-Difference (95%CI)** | **Adjusted p-value** |
| --- | --- | --- | --- | --- | --- |
| Primary Physician Visits | 0·47  (0·47, 0·48) | 0·48  (0·47, 0·48) | 1·01  (1·00, 1·03) | -0·006  (-0·001, 0·013) | 0·29 |
| Specialty Physician Visits | 0·17  (0·16, 0·17) | 0·18  (0·17, 0·18) | 1·04  (1·01, 1·07) | 0·006  (0·0008, 0·011) | 0·12 |
| ER Visits | 0·031  (0·029, 0·033 | 0·034  (0·032, 0·035) | 1·10  (1·01, 1·20) | 0·003  (0·0004, 0·006) | 0·12 |
| Hospital Admissions | 0·008  (0·007, 0·009) | 0·0077  (0·0070, 0·0084) | 1·02  (0·87, 1·20) | 0·0001  (-0·001, 0·001) | 1 |
| Prescriptions | 0·31  (0·30, 0·31) | 0·32  (0·32, 0·32) | 1·04  (1·02, 1·06) | 0·012  (0·005, 0·018) | 0·007 |
| New Diagnoses | 0·015  (0·014, 0·017) | 0·016  (0·015, 0·017) | 1·03  (0·91, 1·17) | 0·0005  (-0·002, 0·002) | 1 |
| Referrals for Mental Health Services | 0·0029  (0·0022, 0·0037) | 0·0041  (0·0036, 0·0046) | 1·45  (1·07, 1·89) | 0·001  (0·0003, 0·002) | 0·05 |

Figure S1 - Age of study participants at the index date.


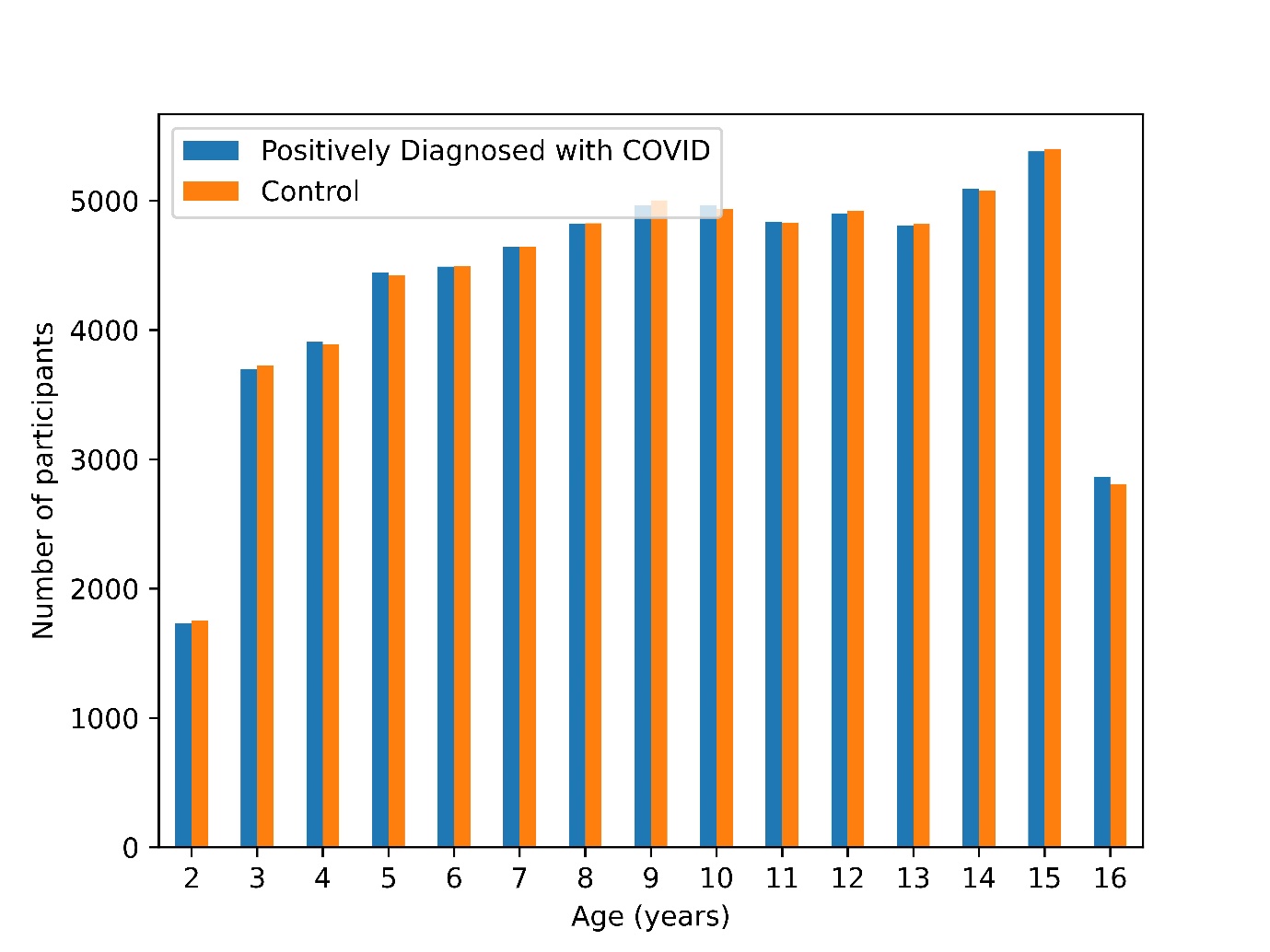


Figure S2 - Distribution of the index date, that is, the day the disease was acquired.


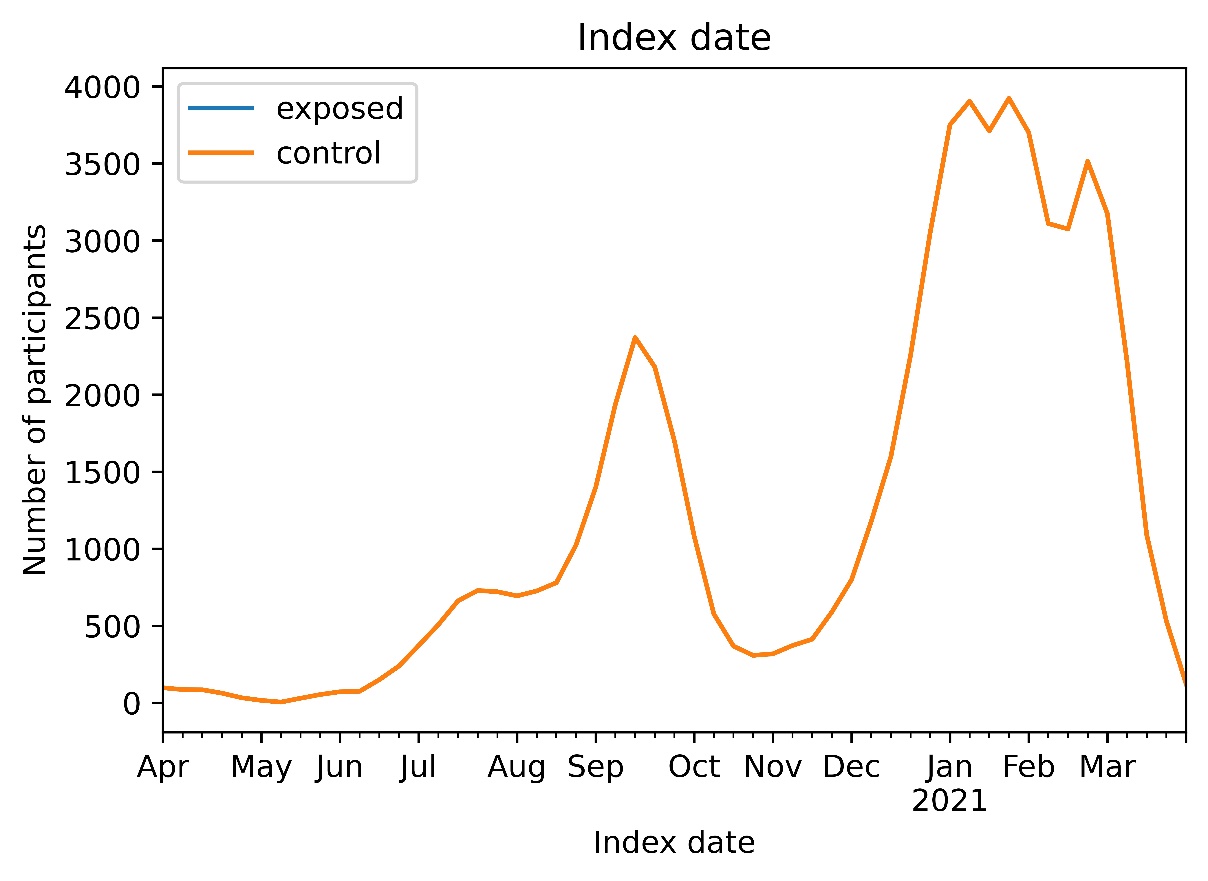


Figure S3 - Unadjusted relative risk for use of different services before and after the index date. In green are the periods long before the index day, in yellow are the periods one month before the index day, in gold are the periods immediately after the index day, in orange are the periods 1-3 month after the index day, and in crimson is the period 3-6 month after the index day.

| 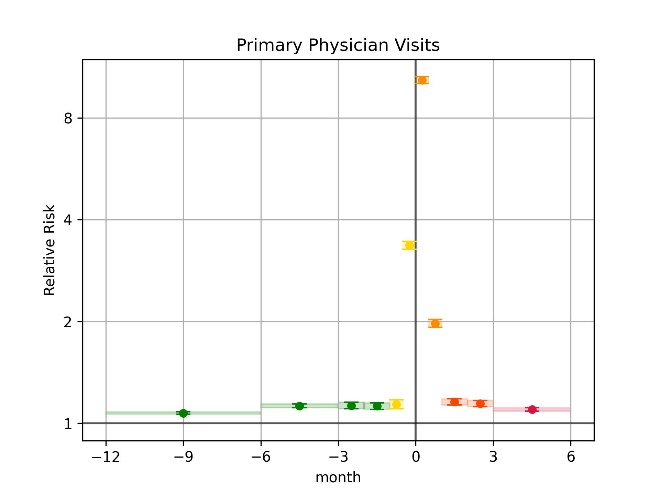 | 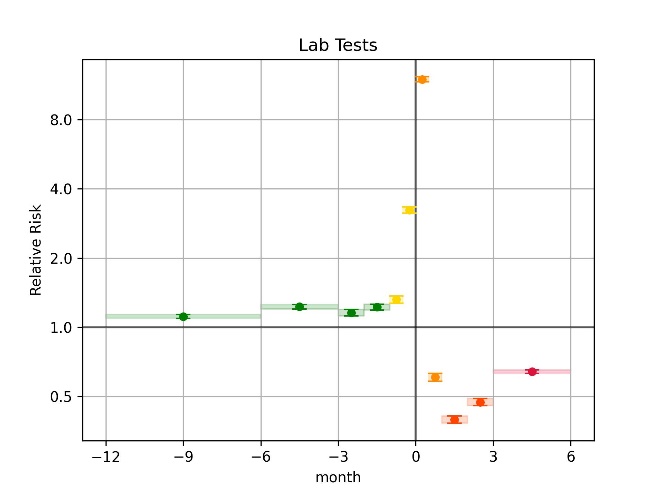 |
| --- | --- |
| 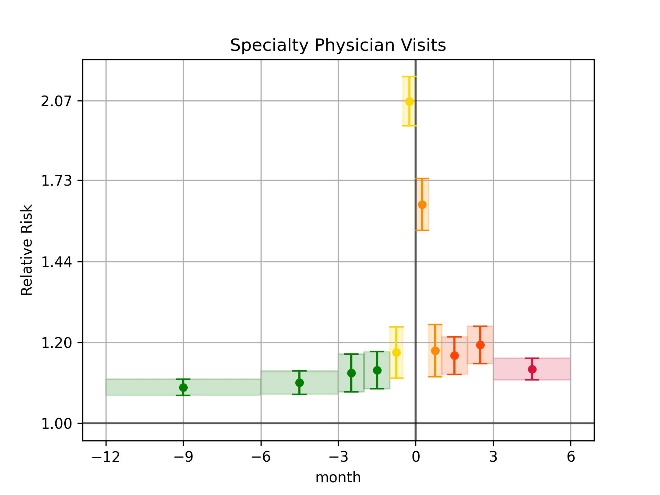 | 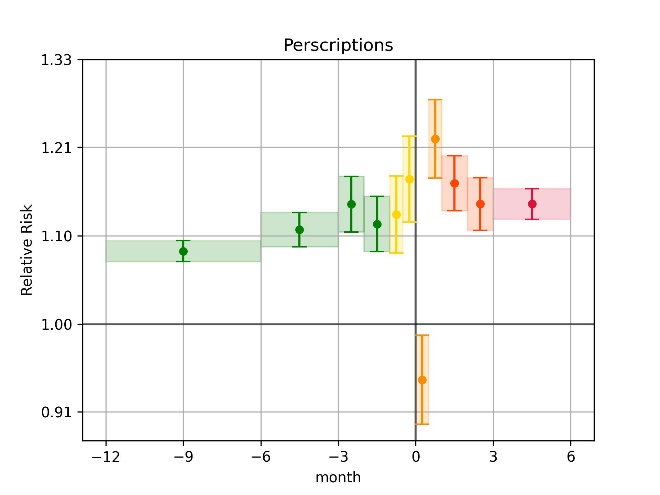 |
| 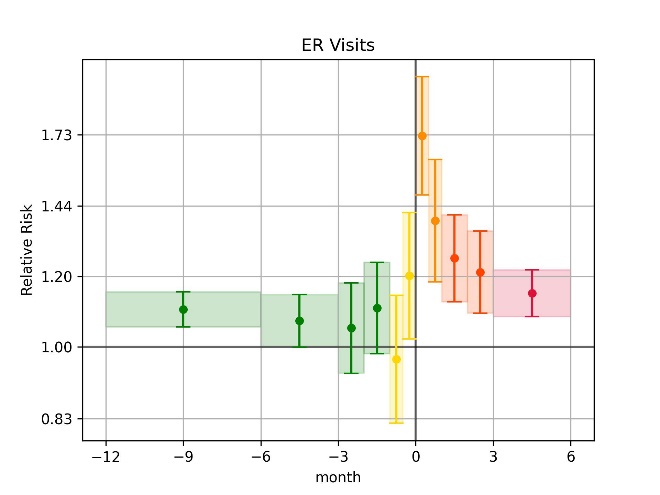 | 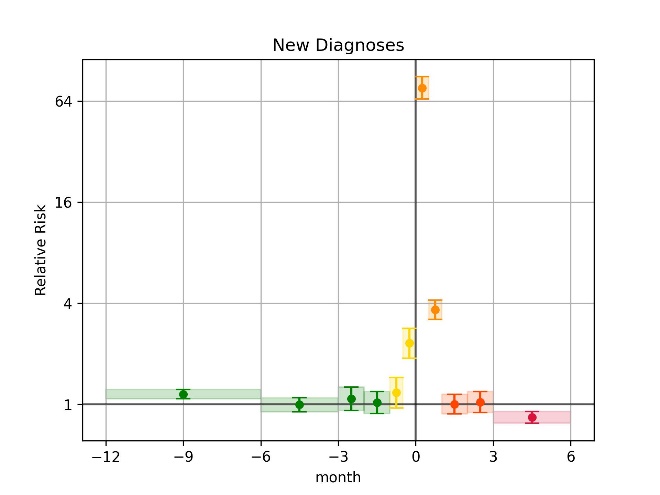 |
| 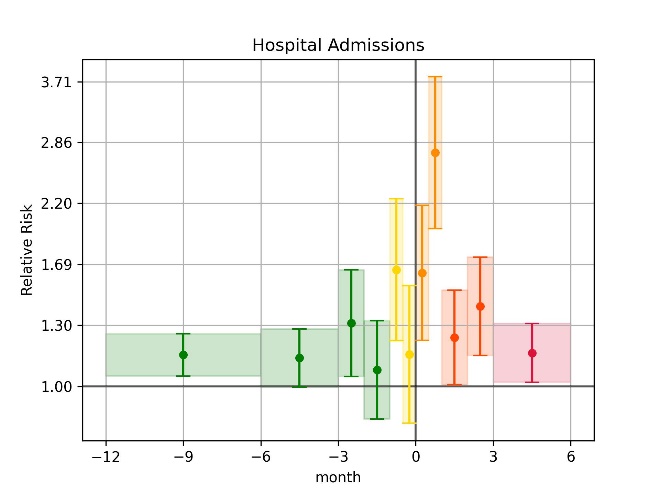 | 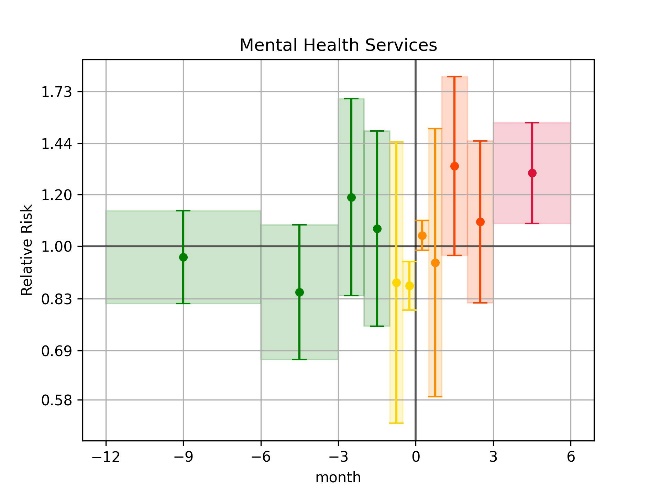 |
